# Early versus interval appendicectomy for localised perforated appendicitis in children: A best evidence review

**DOI:** 10.1016/j.amsu.2020.09.028

**Published:** 2020-09-26

**Authors:** Pushpa Veeralakshmanan, James Ackah, Pedram Panahi, Rashid Ibrahim, Mark Coleman

**Affiliations:** Department of General Surgery, University Hospitals Plymouth, United Kingdom

**Keywords:** Evidence-based medicine, Perforated appendicitis, Early appendicectomy, Interval appendicectomy

## Abstract

A best evidence topic in general surgery was written according to a structured protocol. The question addressed was whether early or interval appendicectomy provides a superior clinical outcome for children presenting with localised perforated appendix. Altogether 204 papers were found using the search strategy reported below; of which 5 were identified to provide the best evidence to answer the question. The author, journal, date and country of publication, patient group studied, study type, relevant outcomes, results, and study weaknesses were tabulated. We concluded that for children presenting with localised perforated appendix without abscess formation, early appendicectomy provides better clinical outcome in terms of lower complication and re-admission rate and shorter length of hospital stay.

## Highlights

• There is controversy in the management of perforated appendicitis in children.
• Limited studies focus on analysing free perforated appendicitis separately from appendicular abscess, mass and phlegmon.
• Localised perforated appendicitis treated with early appendectomy have lower complication and re-admission rate.

## 1 Introduction

A best evidence topic was constructed according to a structured protocol outlined by the International Journal of Surgery [1].

## 2 Clinical scenario

An 11-year-old boy presents with abdominal pain and vomiting. On examination, his right iliac fossa is tender. He has raised inflammatory markers and an ultrasound is performed that shows presence of free fluid around the appendix. You are suspecting acute appendicitis with localised perforation of the appendix. You start initial management of intravenous fluids and antibiotics; but you are unsure whether to consent for immediate appendicectomy or treat him conservatively and perform interval appendicectomy. You decide to check the recent literature for evidence.

## 3 Three part question

In [children with localised perforated appendix without abscess formation], is [early appendicectomy superior to delayed/interval appendicectomy] in terms of clinical outcome [Length of hospital stay, adverse events, re-admission rate].

## 4 Search strategy

Medline ® 1946 to June week 1 2020 and Embase 1974 to 2020 June 08 using OVID interface: [Perforated appendicitis* OR Localised perforation* OR Complicated appendicitis*] AND [Early appendicectomy* OR Interval appendicectomy*] Limit to English.

Medline ® using PubMed interface: [Perforated appendicitis OR Localised perforation OR Complicated appendicitis] AND [Early appendicectomy OR Interval appendicectomy] Limit to Children age 0-18.

## 5 Search outcomes

**Fig. 1:**
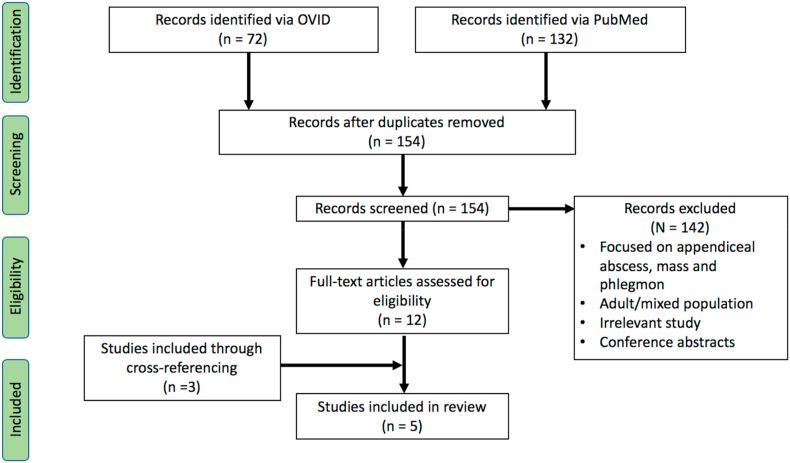
PRISMA flow diagram.

A total of 204 papers were found via OVID and PubMed interface. 192 papers were excluded based on titles, abstract and duplicates. 12 full-text articles were screened and assessed for eligibility. A further 3 papers were included by scanning the references of relevant papers. From these, 5 papers were identified that provided the best evidence to answer the question. This is represented in Fig. 1. The results of the 5 papers included for review are presented in Table 1.

**Table 1:** Best evidence papers.

Author, date, journal and country, study type (level of evidence)	Patient group	Outcomes	Key results	Study Weakness
Blakely M. et al. (2011), Archives of Surgery, USA, Non-blinded prospective RCT, Level II	131 patients Group A – Early Appendicectomy within 24 h of admission (n = 64) Group B – Interval appendicectomy (n = 67)	Time away from normal activities (days)	A: 13.8 (7.5) B: 19.4 (8.7) P < 0.001	Exclusion criteria – discretion of attending pediatric surgeon Single centre study – difficulty with generalisability of findings
		Length of hospitalization (days)	A: 9.0 B: 11.2 P = 0.03	
		Adverse event rate (n, %)	A: 18 (30%) B: 35 (55%) P = 0.003	
Tsai HY. et al. (2016), Journal of Paediatrics and Neonatology, Taiwan, Retrospective observational study, Level III	122 patients Abscess group (ABS) and non-abscess group (NA). Further classified into conservative (CS) and early appendectomy (EA) groups. Focus on NA group for this review. N = 37 NA-CS: 16 NA-EA: 21	Intravenous antibiotics duration (days)	NA-CS: 12.2 NA-EA: 5.5 P < 0.001	Retrospective review – bias in patient selection 6 patients with false positive perforated appendix.
		Length of hospital stay (days)	NA-CS: 12.6 NA-EA: 6.0 P < 0.001	
		Readmission within 1 month, number (n, %)	NA-CS: 2 (12.5%) NA-EA: 2 (9.5%) P > 0.99	
Bufo AJ. Et al (1998), Journal of laparoendoscopic and advanced surgical techniques, USA, retrospective cohort study, Level III	87 patients Group A: Immediate appendectomy (n = 46) Group B: Interval appendectomy (n = 41).	Length of hospital stay (days) mean ± SD	A: 6.1 ± 3.1 B: 5.6 ± 4.0 P < 0.01	Study group also includes patients with localised abscess and phlegmon. ‘failure’ of treatment for patients assigned to interval appendectomy were not considered as surgical complications.
		Number of patients with Surgical complications in each group (n, %)	A: 10 (21%) B: 2 (6%) P < 0.03	
		Hospital charges (USD) mean ± SD	A: 11,044 ± 11,321 B: 6435 ± 4447 P < 0.02	
Weber T. Et al (2003), American Journal of Surgery, USA, prospective cohort study, Level III	96 patients Group 1: Immediate appendectomy (n = 71) Group 2: Successful Interval appendectomy (n = 16) Group 3: Unsuccessful interval appendectomy (n = 9)	Total length of stay in hospital (days)	1: 6.5 2: 9 3: 11 Statistically not significant	Subjective diagnosis of perforated appendicitis Study number for comparison is very small.
		Number of patients with surgical complications (%)	1: 28% 2: 12.5% 3: 11% P < 0.05	
		Complication rate requiring rehospitalization (%) **Group 2 and 3 are combined to give n = 25.	1: 28% 2&3: 12% P < 0.05	
Henry M.C.W. Et al (2007), Journal of Pediatric Surgery, USA, Retrospective multicentre case control study, Level III	313 patients reviewed, further case control matched Control Group: Immediate appendectomy (n = 48) Case Group: Nonoperative management (n = 48)	Surgical complication rate (n, %)	Control: 20, 43% Case: 9, 19% P < 0.01	Data analysis did not include any subsequent hospitalization that occurred for interval appendectomy. Case and control group matched for 12 clinical parameters – no evidence to show the relevance of these 12 clinical parameters to the outcome.
		Postoperative abscess formation rate (n, %)	Control: 11, 24% Case: 2, 4% P < 0.01	
		Length of hospital stay (days, SD)	Control: 8.8 ± 6.7 days Case: 6.5 ± 5.7 days P = 0.08	

## 6 Discussion

Despite children presenting with perforated appendicitis being common, there has been controversy between performing immediate appendectomy and interval appendectomy 6–8 weeks after initial presentation. Although we identified 5 studies relevant to addressing the question, only 2 studies (Blakely et al. and Tsai HY et al.) analysed free perforated appendicitis separately from perforated appendicitis with appendicular abscess, appendicular phlegmon and mass.

Blakely et al. [2] in 2005 performed a non-blinded prospective randomised controlled trial (RCT) on 131 patients in which the primary outcome measured was time away from activities in days. This study favoured early appendectomy as the children with perforated appendix who were treated with early appendectomy returned to normal activities on average of 5 days earlier (P < 0.001). The overall adverse event rate and length of hospital stay was also significantly lower in the early appendectomy group. Most frequent adverse events in the interval group included intra-abdominal abscesses during the treatment period, development of small bowel obstruction and unplanned readmission. Two patients that were assigned to interval appendectomy group didn't have an appendectomy due to failure to return for treatment. The randomisation and method of allocation was adequate and eligibility criteria was clearly defined and intention to treat analysis was performed making this RCT of good quality. Tsai HY et al. [3] performed a retrospective observational study over a 2 year period. Patients were sub-classified into having perforated appendix with no abscess or phlegmon (NA) and presence of abscess or phlegmon (ABS). The NA patients were further sub-classified into conservative treatment (NA-CS) and early appendicectomy (NA-EA). This study favours early appendicectomy in the NA group of patients. NA-EA had significantly shorter duration of stay in the hospital and shorter course of intravenous antibiotics. 6 patients (37.5%) in the NA-CS group required antibiotic escalation and 0 patients in the NA-EA group required antibiotic escalation. The main limitation of this study is that this is a retrospective review and there is an inherent patient selection bias. The study states that the surgeons deferred early appendicectomy in patients with serum CRP level greater than 100 mg/L, which can imply that the EA group may have a milder disease severity compared to the CS group.

The other 3 studies included in this review (Bufo et al., Weber et al. and Henry et al.) included mixed patients with free perforated appendicitis and associated appendiceal abscess, phlegmon or mass. Bufo AJ et al. [4] performed a retrospective cohort study over a 2 year period. The age group in the study ranged from 2 to 18 years. After initial treatment, patients were grouped into either immediate appendectomy or interval appendectomy group. Overall, the study claims to have found a shorter hospital stay, decreased cost, and fewer complications in those patients successfully treated non-operatively (interval appendectomy group). However, In the interval appendectomy group, 7 (17%) patients failed their initial antibiotic therapy and hence required appendectomy on the initial admission but this was not included in their initial analysis. Subsequent intention to treat analysis showed no significant differences in the outcomes between the interval and immediate appendectomy group. Weber T et al. [5] performed a prospective cohort study over a 4 year period. The age group in the study ranged from 2 to 16 years. The diagnosis of perforated appendicitis was made clinically in all patients with 40% undergoing computer tomography (CT) scan to confirm diagnosis and detecting abscesses that may require percutaneous drainage. 74% of the patients (n = 71) underwent immediate appendectomy (Group 1) due to presence of peritonitis with sepsis or large abdominal/pelvic abscesses that were not suitable for percutaneous/operative drainage. The remaining 25 patients who presented with less severity of illness were subject to interval appendectomy. All 25 patients underwent CT scan to detect any abscess that require drainage. It could be argued that the patients in group 1 are clinically different as they are clearly sicker group of patients. This makes reaching a conclusion when comparing the outcomes difficult. 16 patients (Group 2) successfully completed antibiotic course and underwent interval appendectomy in 6–8 weeks. 9 patients (Group 3) remained symptomatic despite antibiotic therapy and required appendectomy (range of 3–12 days after presentation). Although this study classifies patients into three distinct groups, when comparing interval versus immediate appendectomy, out of the 25 patients subject to interval appendectomy, 36% (n = 9) failed medical therapy and required appendectomy earlier. Additionally, 74% (n = 71) patients were in group 1 and only 25 patients were in group 2&3 combined. This can potentially skew data for comparison. For example, length of hospital stay was lowest in Group 1 (6.5 days) compared to 9 and 11 in group 2 and 3 respectively. However, this was not statistically significant possibly due to the patient population in the study being too small. Henry M.C.W et al. [6] performed a retrospective multi-centre case-control study over a 5 year period. The study defined perforated appendicitis as evidence on preoperative abdominal ultrasound or CT scan confirming perforated appendicitis, evidence of perforated appendicitis in the OR, or perforation confirmed on pathology report. Both the case and control groups were clinically matched on 12 clinical parameters to allow for meaningful comparisons of outcomes. This study found that the non-operative group had fewer complications, fewer post treatment recurrent abscesses and a shorter length of stay. Although the length of hospital stay was non-significantly shorter in the non-operative group, this data analysis did not include any subsequent hospitalization that occurred for interval appendectomy. 5 (10.4%) patients failed non-operative management and required appendectomy earlier. These 5 patients had significantly longer hospitalisations with a mean stay of 17.6 ± 12.4 days which was not included in the initial data analysis.

## 7 Clinical bottom line

Most of the studies included in this review are retrospective in nature and are of low quality due to significant selection bias. However, this is understandable as there is difficulty in determining perforation at presentation through clinical examination alone which subsequently has an impact on determining the treatment choice. Additionally, only 2 of these 5 papers reviewed directly address the three part question of managing children presenting with localised perforated appendix without abscess formation. Based on the evidence from the papers reviewed, early appendectomy should be performed in children presenting with localised perforated appendicitis with no appendiceal abscess formation. Children with localised perforation with no abscess formation that are treated with early appendectomy within 24 h of presentation showed lower complication and re-admission rate and shorter length of stay in hospital. Regardless of the choice of surgical treatment, resuscitation with intravenous fluids and broad spectrum antibiotics is agreed upon universally. Presentation with perforated appendicitis with presence of well-formed abscess should be drained either operatively or through interventional radiology.

## Provenance and peer review

Not commissioned, externally peer reviewed.

## Annals of medicine and surgery

The following information is required for submission. Please note that failure to respond to these questions/statements will mean your submission will be returned. If you have nothing to declare in any of these categories then this should be stated.

## Please state any conflicts of interest

The authors have none to declare.

## Please state any sources of funding for your research

No funding required.

## Ethical approval

Not Applicable.

## Consent

Not Applicable.

## Author contribution

Pushpa VEERALAKSHMANAN, MB ChB – Conducted literature search and wrote paper.

James ACKAH, MPharm, BMBS, MRCS– Assisted in literature search and writing of paper.

Pedram PANAHI, MBBS, MRes (Dist) – Assisted in writing of paper.

Rashid IBRAHIM, MD, MRCS – Assisted in writing of paper.

Professor Mark COLEMAN, MB ChB, FRCS (Gen Surg), FRCPSG (Hons), MD – Supervising Consultant.

## Registration of research studies

Not Applicable.

## Guarantor

Pushpa VEERALAKSHMANAN, MB ChB.
